# Relationship between emotional exhaustion and empathy in medical students from monteria - colombia

**DOI:** 10.1192/j.eurpsy.2021.1043

**Published:** 2021-08-13

**Authors:** E.P. Ruiz Gonzalez, A.M. Romero Otalvaro, M.C. Crespi, M.N. Muñoz Argel, J.D. Velez Carvajal

**Affiliations:** 1 Psychology, Universidad Pontificia Bolivariana, Monteria, Colombia; 2 Psychology, Universidad de Buenos Aires, Buenos Aires, Argentina

**Keywords:** empathy, exhaustion, doctors in training

## Abstract

**Introduction:**

Empathy is considered one of the most relevant characteristics in the interaction between the doctor and the patient, highlighting the need to enhance it from the professional training stage. However, some studies have established that high levels of empathy could generate emotional exhaustion (Boujut, Sultan, Woemer & Zenasni, 2012). However, if a certain type of empathy can lead to burnout, it must also be considered that an optimal empathic posture can, on the contrary, relieve stress and exhaustion.

**Objectives:**

Establish the relationship between the level of emotional exhaustion and empathy in medical students.

**Methods:**

A cross-sectional study of correlational scope was conducted in 182 (n = 90) medical students. The cognitive and affective empathy test (López, et al., 2008) and the adaptation of the MBI instrument for the Colombian population (Barbato, Córdoba, González, Martínez & Tamayo, 2011) were used to assess emotional exhaustion

**Results:**

Statistically significant correlations of positive magnitude were evidenced between the variables emotional exhaustion and cognitive empathy (Table 1)

**Conclusions:**

It was possible to conclude that the higher levels of cognitive empathy (adoption of perspective) in medical students, also resulted in greater emotional exhaustion, revealing an inappropriate consequence of empathy, where professionals can excessively adopt the patient’s feelings, generating wear. It is essential to promote optimal levels of empathy, which are beneficial for both the patient and the doctor.

